# The CCR5 Gene Edited CD34^+^CD90^+^ Hematopoietic Stem Cell Population Serves as an Optimal Graft Source for HIV Gene Therapy

**DOI:** 10.3389/fimmu.2022.792684

**Published:** 2022-03-14

**Authors:** Karthik V. Karuppusamy, John Paul Demosthenes, Vigneshwaran Venkatesan, Abisha Crystal Christopher, Prathibha Babu, Manojkumar K. Azhagiri, Annlin Jacob, Veena Vadhini Ramalingam, Sumathi Rangaraj, Mohankumar Kumarasamypet Murugesan, Srujan Kumar Marepally, George M. Varghese, Alok Srivastava, Rajesh Kannangai, Saravanabhavan Thangavel

**Affiliations:** ^1^ Centre for Stem Cell Research (CSCR), A Unit of InStem Bengaluru, Vellore, India; ^2^ Manipal Academy of Higher Education, Manipal, India; ^3^ Department of Clinical Virology, Christian Medical College, Vellore, India; ^4^ Thiruvalluvar University, Vellore, India; ^5^ Department of Infectious Diseases, Christian Medical College, Vellore, India; ^6^ Department of Hematology, Christian Medical College, Vellore, India

**Keywords:** CCR5, hematopoietic stem cells, gene therapy, gene editing, HIV, long-term engraftment

## Abstract

Transplantation of allogenic hematopoietic stem and progenitor cells (HSPCs) with C-C chemokine receptor type 5 (CCR5) Δ32 genotype generates HIV-1 resistant immune cells. CCR5 gene edited autologous HSPCs can be a potential alternative to hematopoietic stem cell transplantation (HSCT) from HLA-matched CCR5 null donor. However, the clinical application of gene edited autologous HSPCs is critically limited by the quality of the graft, as HIV also infects the HSPCs. In this study, by using mobilized HSPCs from healthy donors, we show that the CD34^+^CD90^+^ hematopoietic stem cells (HSCs) express 7-fold lower CD4/CCR5 HIV receptors, higher levels of SAMHD1 anti-viral restriction factor, and possess lower susceptibility to HIV infection than the CD34^+^CD90^-^ hematopoietic progenitor cells. Further, the treatment with small molecule cocktail of Resveratrol, UM729 and SR1(RUS) improved the *in vivo* engraftment potential of CD34^+^CD90^+^ HSCs. To demonstrate that CD34^+^CD90^+^ HSC population as an ideal graft for HIV gene therapy, we sort purified CD34^+^CD90^+^ HSCs, treated with RUS and then gene edited the *CCR5* with single sgRNA. On transplantation, 100,000 CD34^+^CD90^+^ HSCs were sufficient for long-term repopulation of the entire bone marrow of NBSGW mice. Importantly, the gene editing efficiency of ~90% in the infused product was maintained *in vivo*, facilitating the generation of CCR5 null immune cells, resistant to HIV infection. Altogether, CCR5 gene editing of CD34^+^CD90^+^ HSCs provide an ideal gene manipulation strategy for autologous HSCT based gene therapy for HIV infection.

## Introduction

Human immunodeficiency virus-1 (HIV-1) infection leads to acquired immunodeficiency syndrome (AIDS) and more than 30 million people are affected by it worldwide (1). Conventionally, viral replication in patients is suppressed by lifetime anti-retroviral therapy (ART). However, long term ART is associated with risks such as drug resistance, immunological non respondence, organ damage and age-related health complications (2, 3). In addition, interruption of ART leads to HIV-1 rebound in patients.

HIV infects the immune cells through the receptors such as CD4, CCR5 and CXCR4, which are predominantly expressed on the host immune cells such as T cells, macrophages and dendritic cells (4). These receptors are the potential targets to block HIV invasion and destruction of critical immune cells (5, 6). Particularly, the CCR5 receptor is an attractive target owing to a naturally occurring CCR5 null variant (CCR5 delta32/delta32), which confers resistance to R5-tropic HIV-1 (7). HSPCs from CCR5 null homozygous individuals have been shown to impart functional cure to AIDS patients on allogeneic transplantation (7, 8). Importantly, CCR5 receptor is dispensable for survival and function of immune cells (9). The limited availability of HLA matched CCR5 null donors, more specifically in African and south-Asian populations with high prevalence of HIV, poses major challenge to this approach as a regular therapy (10).

To this end, gene editing tools such as Zinc Finger Nucleases (ZFN), Transcription activator like effector nucleases (TALEN) and Clustered regularly interspaced short palindromic repeats/CRISPR associated protein 9 (CRISPR/Cas9) provide alternate option for generating CCR5 edited immune cells (4). CCR5 gene editing in CD4 T cells demonstrated the protection of edited cells from HIV mediated T cell lysis in the patients (11, 12). HIV-1 resistance was also reported using CCR5 edited HSPCs in mice models (13). Although both strategies were proved safe in clinical studies, they failed to provide functional cure (14).

Identifying the ideal target cells for CCR5 gene-editing is a key step towards the success of HIV gene therapy. CCR5 gene editing in CD4 T cells protects only the T cells and harvesting high quantity and quality of CD4 T cells from the patients can be a challenge (15). In addition, cellular senescence may deplete the frequency of modified T cells *in vivo* and may require repetition of the procedure (16, 17). Though CCR5 gene editing in HSPCs can generate CCR5 null lineages, it is also associated with certain challenges (4). Unlike, gene manipulation of autologous HSPCs for genetic disorders, HSPCs obtained from infectious disease patients may also contain infectious particles. The presence of HIV pro-viral DNA present an added risk of viral activation during *ex vivo* culture and gene editing procedure (18, 19). The recent non-human primate model transplantation studies have clearly defined the immunophenotypic definition of HPCs and HSCs as CD34^+^CD90^-^ and CD34^+^CD90^+^ cells, respectively but how HIV infects any of these defined subpopulation remains elusive (20). There are also considerable hurdles associated with HSPC gene therapy that can negatively affect the outcome of the manipulation, such as, low frequency of HSCs in the HSPC graft, its reduction during *ex vivo* culture and gene editing, lack of bi-allelic gene editing in HSCs, the need to manipulate large number of HSPCs and the drop in gene editing efficiency post transplantation (4).

In this study, we show that CD34^+^CD90^+^HSC fraction of HSPCs have reduced CD4/CCR5 receptors and increased antiviral restriction factors to limit the HIV-1 infection and demonstrate that they are the potential target cells for CCR5 gene editing. We also show that 100,000 CD34^+^CD90^+^HSCs are sufficient to repopulate the entire mouse bone marrow in order to generate CCR5 null immune cells.

## Materials and Methods

### TZM-bl Cell Lines

TZM-bl cells (JC53BL-13 derived from HeLa, Cat No: ARP-8129) were received through the NIH AIDS Reagent Program, (Division of AIDS, NIAID, NIH). The cells were cultured in DMEM medium with 10% FBS and 1X antibiotic and antimycotic solution (Cat No:15240062, Thermo Fisher scientific). Cells were cultured for at least two passages prior to gene-editing. The HIV infection in TZM-bl was measured by luciferase reporter expression which is under the control of HIV-Tat regulatory element (21).

### Purification of CD34^+^HSPCs, CD34^+^CD90^+^ HSCs and CD34^+^CD90^-^ HPCs

Granulocyte colony stimulating factor (G-CSF) mobilized peripheral blood was collected from healthy donors after obtaining approval from Institutional Review Board (IRB). Peripheral blood mononuclear cells (PBMNCs) were separated using ficoll density gradient centrifugation. CD34^+^ cells were isolated from PBMNCs using EasySep CD34 positive selection kit (Cat No: 17896, STEMCELL Technologies) as per the manufacturer instructions. The purified CD34^+^ cells were pre-stimulated with Serum free essential medium (SFEM) containing hematopoietic stem cell specific cytokines such as SCF (240ng/ml), FLT3 (240ng/ml), TPO (80ng/ml) and IL-6 (40ng/ml) and small molecule cocktail of Resveratrol, Stem Reginin-1 and UM729 (RUS) as described in our previous work (22). All the donors were screened for delta32 genotype before conducting CCR5 gene editing (**Supplementary Figure 3A**). For CD34^+^CD90^+^ and CD34^+^CD90- cell sorting, the purified CD34^+^ cells were briefly cultured with above mentioned cytokines (6-12 hours) and stained with CD90 antibody (8µg/1x10^6^ cells) for 20 min at room temperature. After brief washes with PBS, CD90^+^ and CD90^-^ cells were sorted using BD FACS Aria III in purity mode. The purity of sorted cells was re-assessed by staining with CD34 and CD90 antibodies.

### Transwell Migration Assay

0.1 million HSCs were seeded on the upper chamber of the transwell plate with 100µl of basal medium and the lower chamber was loaded with 500µl of SFEM II cytokines (SCF (240ng/ml), FLT3 (240ng/ml), TPO (80ng/ml) and IL-6 (40ng/ml) medium containing 100 ng/mL of SDF-1α ligand. The plate was incubated at 37°C for 24 hours. On the next day, cells in the lower chamber were counted using trypan blue dye exclusion method. The counts were normalized with non-SDF-1α containing media. Percentage of migration was calculated with the number of cells in the bottom chamber divided by number of cells placed in the upper chamber.

### Colony Forming Units (CFU) Analysis

Based on the experimental requirements, 300 to 500 HSCs were seeded in 3ml of semi-solid methylcellulose medium (Cat No: #04044, Stem cell technologies). After 14-16 days, hematopoietic progenitor colonies were enumerated under microscope. Based on the morphology, colonies were categorized as Burst forming unit-erythroid (BFU-E), Colony forming unit-erythroid (CFU-E), Colony forming unit-granulocyte-monocyte progenitor (CFU-GM) and Colony forming unit- granulocyte, erythrocyte, monocyte, megakaryocyte (CFU-GEMM).

### RNA Isolation and Real-Time PCR Analysis of Antiviral Restriction Factors

Total RNA was isolated from sort purified HPCs and HSCs using RNeasy mini kit (Cat No:74104, Qiagen, Hilden, Germany) as per the manufacturer’s instructions. 1µg of total RNA was reverse transcribed into cDNA using PrimeScript 1^st^strand cDNA synthesis kit (Cat No: 6110A, Takara) and analyzed by Real-Time PCR (RT-PCR) with TB Green *Premix Ex Taq* ™II (Cat No: #RR820A, Takara) as per the manufacturer instructions. RT-PCR data were analyzed using the standard 2^−ΔΔCT^ method and presented as the fold expression normalized to the reference gene Ubiquitin C. Primers used for RT-PCR are listed in **Supplementary Table 2**.

### Purification and Culture of CD4 Cells

CD4 cells were isolated from peripheral blood of the healthy donor using CD4 positive selection kit (Cat No: #17852, stem cell technologies) as per the manufacturer’s instructions and the purity was analyzed using CD3 and CD4 FACS antibodies. Isolated cells were cultured using human XF T cell expansion medium (Cat No: 10981, stem cell technologies) along with CD3/CD28/CD2 T cell activator cocktail (Cat No: 10970, Stem cell technologies). CCR5 gene editing was performed on day 4 of expansion.

### CCR5 Gene-Editing

The two sgRNAs targeting CCR5 gene (E2C5- UGACAUCAAUUAUUAUACAU (GRCh38.p10, Chromosome 3- 46372915 – 46372973), E3C5-CAGCAUAGUGAGCCCAGAAG (GRCh38.p10, Chromosome 3- 46373133 – 46373191) were designed based on the common hits identified using Synthego (https://design.synthego.com/#/), CHOPCHOP (https://chopchop.cbu.uib.no/) and benchling (https://www.benchling.com/crispr/) tools. Cas9-Ribonucleoprotein (RNP) was electroporated using Lonza 4D nucleofector with program DZ100 for HSCs, CN114 for TZM-bl cells and DN100 for CD4 cells. After 72 hours, cells were genotyped by sanger sequencing. The chromatographs obtained from the sequencing were analyzed by Inference of CRISPR Edits (ICE) tool from Synthego (https://ice.synthego.com/#/) The primers used for the region-specific amplification is listed in **Supplementary Table 2**. The deletion induced by dual sgRNA system was analysed using gap PCR and quantified with ImageJ software (https://imagej.nih.gov/ij/download.html).

### Macrophage Differentiation

Macrophage differentiation of HSCs was carried out using the published protocol with minor modifications (23). Briefly, control and edited HSCs were plated in non-tissue culture treated polystyrene plates with macrophage differentiation medium (SFEM-II, SCF (100ng/ml), Flt3-L (50ng/ml), IL-6 (10ng/ml), IL-3 (10ng/ml), GM-CSF (10ng/ml) and M-CSF (10ng/ml). Non-adherent cells were collected every 72 hours and reseeded in the macrophage differentiation medium. Adherent cells were cultured using RPMI medium containing 10% FBS along with GM-CSF (10ng/ml) and M-CSF (10ng/ml). After 14-16 days, adherent cells were observed under microscope for morphology, harvested with Accutase (Material Number: 561527, BD Biosciences), stained for CD4 PE, CD14 FITC, CCR5 APC, CXCR4 APC, CD14 BV421, CD80 FITC, CD206APC, CD64 PE, CD163-PE CF594 and CD71 FITC antibodies and analyzed using BD FACS Aria III flow cytometer. Phagocytic potential of generated macrophages was validated using pHrodo Red *E. coli* BioParticles conjugate (Cat No, P35361, Thermo scientific) as per the manufacturer’s instructions. The proportion of phagocytosis was calculated by enumerating the phagocytosis positive and negative cells.

### HIV Production and Challenge Assay

All the HIV related experiments were carried out under BSL-2 facility using BSL-3 practices after the approval of Institute Biosafety committee. HIV-1 p49.5 R5-tropic molecular clone (Cat No: ARP-11389) was obtained through NIH AIDS Reagent Program, Division of AIDS, NIAID, NIH. The clone was contributed by Dr. Bruce Chesebro. For HIV production, plasmid was transfected into HEK293T cells using standard calcium phosphate method. 72 hours of post transfection, media containing viral particles was collected and filtered with 0.45µm filter and stored at -80°C as multiple aliquots. HIV production was monitored by measuring p24 antigen using ELISA kit, obtained from R&D biosystems (Cat No: DHP240B). Infectivity of the HIV-1 stock was determined using TZM-bl cells. HIV challenge assay with gene edited TZM-bl cells and macrophages was done using 25ng and 150ng of p24 respectively for 6 hours in growth medium containing polybrene (8µg/ml). HIV infectivity assay in HSPCs and its subsets were performed as described (24). In brief, 0.25 million HSPCs/HPCs/HSCs were seeded in 250µl of SFEM-II medium (with 8µg/ml of polybrene) in 48-well Retronectin coated plates and R5-tropic HIV-1 (200ng of p24) was added. The plate was subjected to spinfection for 30 mins at 900g and incubated at 37°C for overnight with 5% CO_2_. On the next day, cells were washed with PBS by centrifugation, cultured for 4 to 6 days and stained with HIV Gag antibody. (KC 57- FITC, Beckman coulter).

### HIV Proviral DNA Amplification

Total genomic DNA was isolated from equal number of infected and non-infected HSPCs/HPCs/HSCs and subjected to PCR amplification using HIV specific primers listed in **Supplementary Table 2**. The PCR was conducted using HotstarTaq master mix as per the standard PCR protocol using 50ng of DNA template.

### NBSGW Transplantation Studies

All animal experiments were conducted after obtaining approval from institute animal ethical committee, Christian Medical College, Vellore, Tamil Nadu, India. The Nonirradiated NOD, B6. SCID Il2ry^-/-^ Kit W^41/41^ (NBSGW) mice (Jackson laboratory) were bred in inhouse animal facility. Depending on the experimental requirements, 0.5-1x10^5^ gene-edited HSCs were infused into busulfan conditioned 7-8 weeks old female NBSGW mice *via* tail vein injection. After 16 weeks of infusion, mouse bone marrow, peripheral blood and splenic cells were harvested and analysed for human cell engraftment using human CD45 and mouse CD45.1 antibodies. The percentage of human cell engraftment was calculated using the formula: = (% hCD45)/(% hCD45 + % mCD45.1) x 100. The gene editing in the engrafted cells was analysed by extracting the DNA using Quick DNA Extract (Cat No: QE0905T, Lucigen) and subjected for sequencing with human CCR5 specific primers and ICE analysis. Multilineage repopulation was analyzed with lineage specific antibodies listed in **Supplementary Table 1**.

### Statistical Analysis

All the statistical analysis was performed using PRISM GraphPad 8 package (GraphPad Software Inc., San Diego, CA, USA). Data analysis was done using two tailed unpaired t-test and multiple T-test using Holm-sidak method as indicated in figure legends. Error bars denotes ± SEM. Number of independent experimental replicates (n), number of donors used are indicated in the figure legend. *P* value < 0.05 is considered as statistically significant.

## Results

### Mutagenesis by CRISPR-Cas9 InDels Generates HIV-1 Resistance Similar to Deletion of CCR5 Coding Region

**Figure 1:**
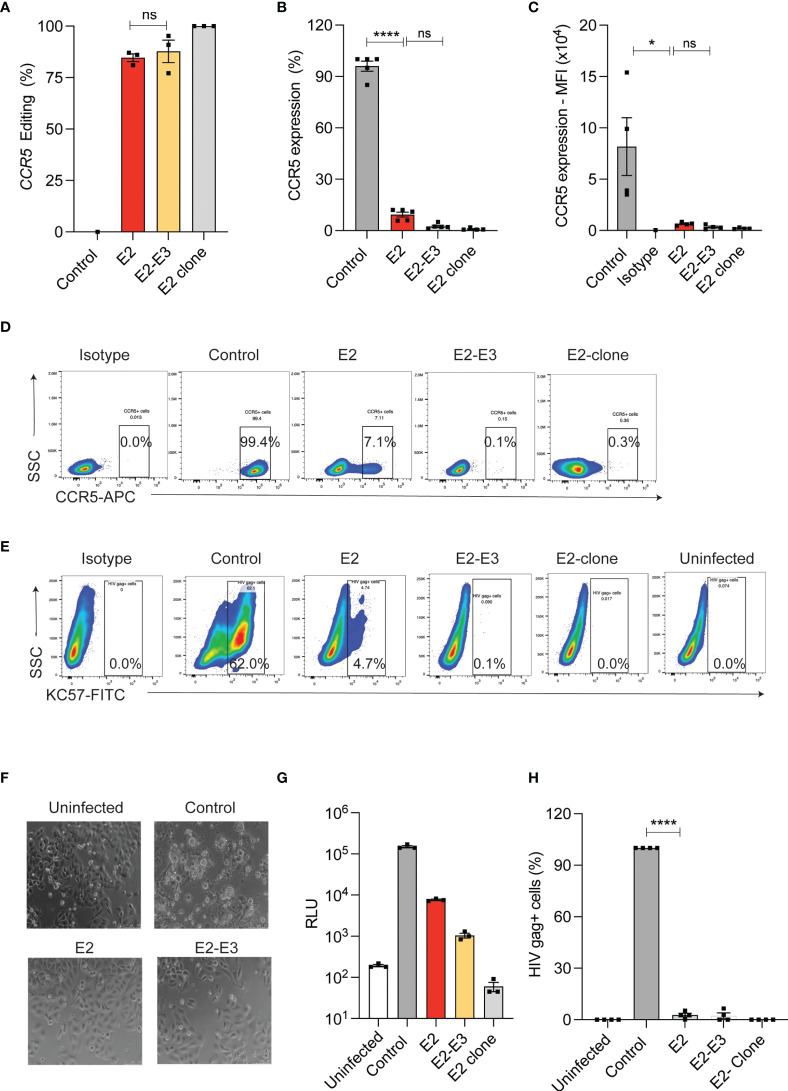
InDel mutagenesis in *CCR5* provides HIV resistance in TZM-bl cells. **(A)** Percentage of *CCR5* gene-editing with single (E2) and dual (E2, E3) sgRNA in TZM-bl cells. (n = 3). E2-clone: homozygous InDel clonal cell line generated from E2-sgRNA edited TZM-bl cells. **(B)** Percentage of CCR5 expression in control and *CCR5* gene-edited TZM-bl cells. CCR5 expression was analyzed by flowcytometry (n = 5). **(C)** Mean Fluorescence Intensity (MFI) of CCR5 in control and CCR5 edited TZM-bl cells (n = 4). **(D)** Representative FACS plot showing CCR5 expression in control and CCR5 gene-edited TZM-bl cells. **(E)** Representative FACS plot showing HIV Gag+ cells in control and CCR5 gene-edited TZM-bl cells after 48 hours of HIV infection. **(F)** Representative phase contrast micrograph of control and CCR5 gene-edited TZM-bl cells at 10x magnification. TZM-bl cells were infected with 25ng of HIV and 48 hours post infection, the cell morphology was analysed. Scale bars were indicated at the right corner of the image. **(G)** Luciferase expression measured as relative light unit (RLU) after 48 hours of HIV infection in control, CCR5 edited TZM-bl cells, (n = 3). **(H)** Percentage of HIV gag positive cells in control and CCR5 edited TZM-bl cells. (n = 4). HIV infection (Gag+ cells) in control TZM-bl cells was normalized to 100. Error bars denotes mean ± SEM, ns; non-significant. *p ≤ 0.05, ****p ≤ 0.0001. Statistical analysis was performed using multiple t-test (holm-Sidak method).

Naturally occurring deletion (Δ32) in the coding region of CCR5 is shown to ablate CCR5 expression. Generating CRISPR-Cas9 mediated CCR5 Δ32bp genotype or deletion of coding region in HSPCs needs incision by two sgRNAs of similar efficiency and this procedure increases the chance of off- target editing and chromosomal rearrangements (13, 25, 26). To test the impact of small InDels on CCR5 expression, we used single sgRNA (E2) that targets coding region and compared it with dual sgRNA (E2 and E3) approach that deletes 246 bp in TZM-bl cells (**Supplementary Figure 1A**). High frequency of InDels (>80%) with single sgRNA and deletion (>85%) with dual sgRNA were detected by ICE analysis of Sanger sequencing reads and by gap PCR, respectively (**Figure 1A**). Both the single sgRNA and dual sgRNA approaches resulted in reducing CCR5 expression to <12% and <3%, respectively, compared to un-edited control. (**Figures 1B–D**).

Next, we challenged the single and dual sgRNA edited TZM-bl cells with R5-tropic HIV. While the control cells showed cell death (**Figure 1F**), and high frequency of HIV infection as indicated by luciferase reporter expression (**Figure 1G**) and by intracellular HIV gag p24 staining (**Figures 1E, H**), all the edited conditions showed complete HIV resistance. These observations suggest that single sgRNA mediated InDels are sufficient to provide HIV resistant phenotype similar to deletion of CCR5 coding region.

### Limited Expression of CD4/CCR5 Receptors on CD34^+^CD90^+^ HSCs Contributes to the Reduced Susceptibility for R5-Tropic Infection

**Figure 2:**
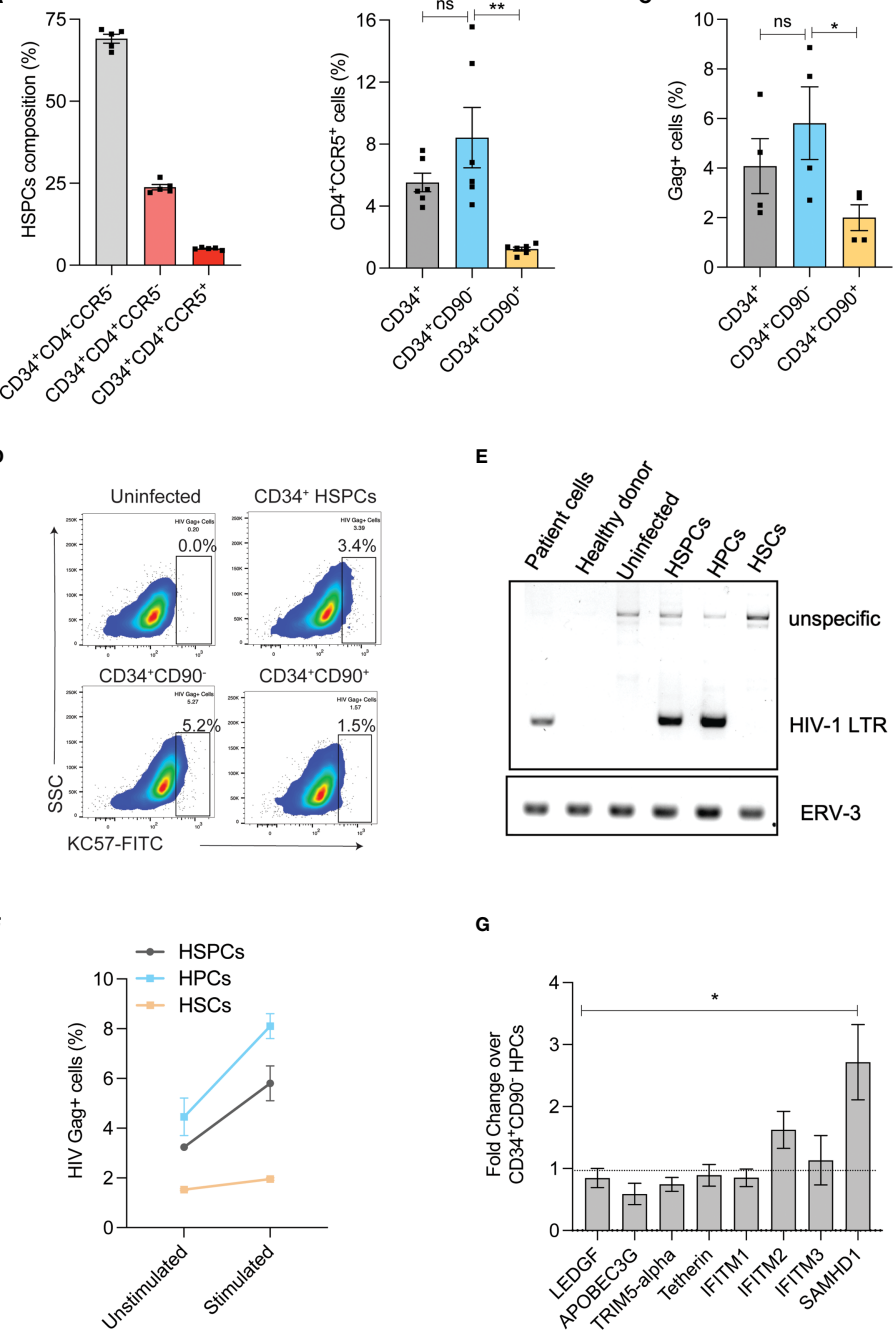
HIV-1 infection in HSPC sub-populations. **(A)** Expression of HIV-1 receptors in HSPCs. Flow cytometric analysis of HIV receptors was conducted immediately after the purification of HSPCs (uncultured cells). (n = 5), Donors: 3. **(B)** Percentage of CD4^+^CCR5^+^ cells in CD34^+^ HSPCs, CD34^+^CD90^-^ HPCs and CD34^+^CD90^+^ HSCs. (n = 6), Donors: 3. **(C)** Percentage of HIV-1 infection in CD34^+^ HSPCs, CD34^+^CD90^-^ HPCs and CD34^+^CD90^+^ HSCs. Cells were infected with 200ng of R5-tropic HIV and 7-days post infection, intracellular flowcytometric staining of Gag+ cells were analyzed. (n = 4), Donors: 2. Error bars denotes mean ± SEM, *p ≤ 0.05 (Unpaired t-test, Two-tailed). **(D)** Representative FACS plot showing HIV Gag+ cells in CD34^+^HSPCs, CD34^+^CD90^-^HPCs and CD34^+^CD90^+^ HSCs, 7 days post HIV infection. **(E)** Agarose gel electrophoresis image showing PCR analysis of HIV proviral DNA in CD34^+^ HSPCs, CD34^+^CD90^-^ HPCs and CD34^+^CD90^+^ HSCs. The genomic DNA was extracted from HIV-1 patient PBMNCs, healthy individual cells, uninfected HSPCs and HIV-1 infected cells, and analysed with primers that amplify a region covering HIV-1 LTR and gag (Labelled as LTR), the PCR also produced an unspecific band at 1.5kb. Human endogenous retroviral sequence ERV3 used as a loading control. (n = 2), Donor: 2. **(F)** Viral outgrowth from HIV infected CD34^+^ HSPCs, CD34^+^CD90^-^ HPCs and CD34^+^CD90^+^ HSCs and before and after stimulation with monocyte differentiation medium for 7-days. Percentage of HIV-1 infection was measured by intracellular flowcytometric staining of Gag+ cells. (n = 2), Donor: 1. **(G)** Expression levels of antiviral restriction factors in CD34^+^CD90^+^HSCs compared to CD34^+^CD90^-^ HPCs, as measured by Real-time PCR analysis (Ubiquitin-C is used as internal control). (n = 4) Donors: 4. Error bars denotes mean ± SEM, *p ≤ 0.05, **p ≤ 0.01. [Statistical analysis was performed using multiple t-test (holm-Sidak method)]. ns, non-significant.

HIV infection in HSPCs has been reported and this can potentially limit the use of autologous HSPCs for CCR5 gene editing as the transplanted cells can later serve as reservoir for HIV infection (27, 28). HIV receptor-CD4 and co-receptor-CCR5 are crucial for R5-tropic HIV infection and therefore, we examined the expression of CD4/CCR5 on the G-CSF mobilized HSPCs from the healthy donors. In agreement with previous findings (29, 30), HSPCs expressed HIV receptors. While 25% of HSPCs expressed only CD4 (CD34^+^CD4^+^CCR5^-^ cells) around 6% of HSPCs had both the receptors (CD34^+^CD4^+^CCR5^+^ cells) (**Figure 2A** and **Supplementary Figure 1C**). To identify the subpopulation of HSPCs that expresses both CD4 and CCR5 (CD4/CCR5) receptors, we sorted HSPCs as CD34^+^CD90^-^ HPCs and CD34^+^CD90^+^ HSCs (31) (**Supplementary Figure 1B**). The CD34^+^CD90^+^ HSCs contained 7-fold reduced CD4^+^CCR5^+^ receptors than CD34^+^CD90^-^ HPCs (8.4% *vs* 1.2%) (**Supplementary Figure 1C** and **Figure 2B**).

To validate this finding, we infected the CD34^+^ HSPCs, CD34^+^CD90^-^ HPCs and CD34^+^CD90^+^ HSCs with R5-tropic HIV. Consistent with the pattern of HIV receptor expression, the CD34^+^CD90^+^ HSCs displayed lesser HIV gag p24 staining than the HPCs and HSPCs (3-fold and 2-fold, respectively) (**Figures 2C, D**). Next, on examination of HIV-1 proviral DNA integration, the HSPCs and CD34^+^CD90^-^ HPCs had proviral DNA but not the CD34^+^CD90^+^ HSCs (**Figure 2E**). To further confirm that CD34^+^CD90^+^ HSC fraction effectively resist R5- tropic HIV infection, HIV infected HSPCs, HPCs and HSCs were cultured in monocyte stimulation medium for 7 days. While, both HSPCs and CD34^+^CD90^-^ HPCs had 2-fold increase in viral outgrowth post-stimulation, such increase was not detected in CD34^+^CD90^+^ HSCs (**Figure 2F**).

The presence of high levels of antiviral restriction factors have shown to restrict HIV-1 infection in resting CD4 T cells (32–34). To examine whether such phenomenon exists in the CD34^+^CD90^+^ HSCs, we investigated the expression of anti-viral restriction factors. Real time PCR quantification indicated the high expression of IFITM2, and SAMHD1 in the CD34^+^CD90^+^ HSC fraction when compared with the CD34^+^CD90^-^ HPCs. In particular, SAMHD1 which is shown to restrict HIV infection (33) was expressed 2.5-fold higher in CD34^+^CD90^+^ HSCs (**Figure 2G**). All these findings suggest that reduced expression of HIV receptors and presence of higher levels of antiviral restriction factors are mediating the resistance of CD34^+^CD90^+^ HSCs to HIV infection. These findings also suggest that CD34^+^CD90^+^ HSCs are ideal target cells for CCR5 gene manipulation for HIV gene therapy.

### The Engraftment Potential of CCR5 Edited CD34^+^CD90^+^ HSCs Is Augmented by RUS Treatment

Recent study showed that the gene editing of BCL11A binding site in CD34^+^CD90^+^ HSCs reduced the requirement of target cell population by 10-fold for gene-manipulation and resulted in durable engraftment in non-human primates (35). We reported that sort enriched CD34^+^CD90^+^ HSCs can be preserved with a small molecule cocktail of Resveratrol, UM-729 and SR-1 (RUS) (22).

**Figure 3:**
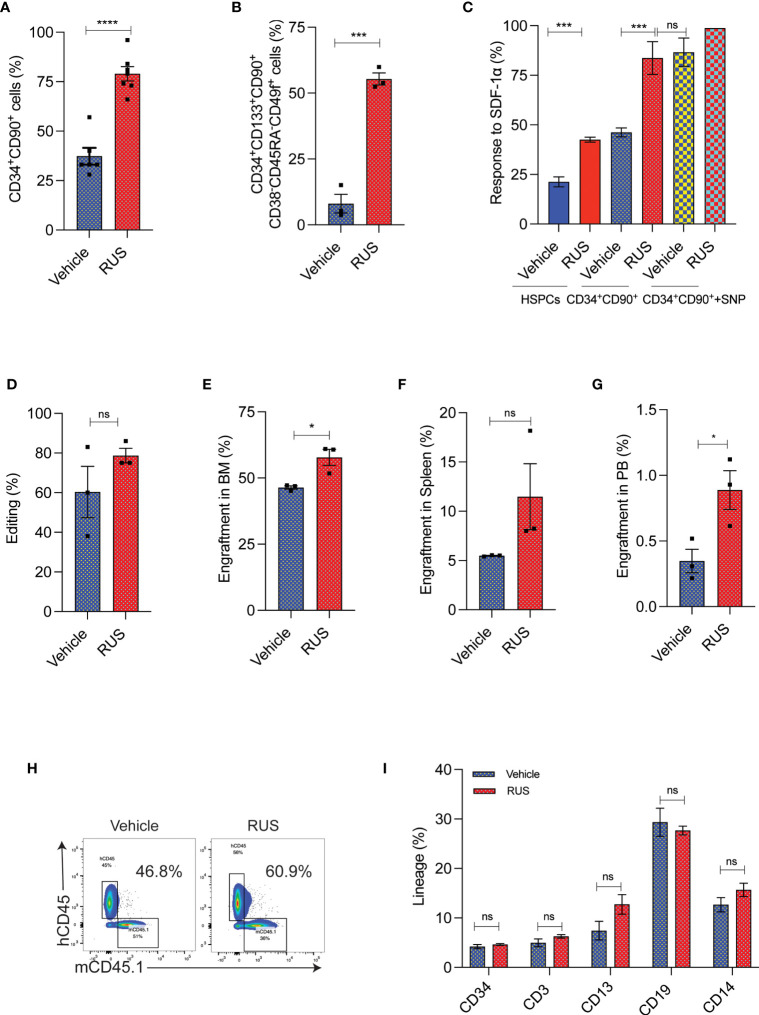
RUS treatment improves the engraftment potential of *CCR5* gene edited CD34^+^CD90^+^ HSCs. Experimental outline for **(A, D)** The FACS purified CD34^+^CD90^+^ HSCs were cultured with vehicle or RUS for 48 hours and subjected for CCR5 editing with 50pM of RNP. Cells were collected 24 hours post editing for the following analysis. Error bar denotes mean ± SEM. ns; non-significant. ***p ≤ 0.001, ****p ≤ 0.0001 (Unpaired t test, two-tailed). **(A)** Percentage of CD34^+^CD90^+^ cells as analysed by flow cytometry. (n = 7), Donors: 4. **(B)** Percentage of CD34^+^CD133^+^CD90^+^CD38^-^CD45RA^-^CD49f^+^ cells as analysed by flow cytometry. (n = 3), Donors: 2. **(C)** Percentage of HSCs responding to SDF1-α in the lower chamber in the transwell migration analysis. The CD34^+^CD90^+^+SNP group was treated with 10µM of Sodium Nitroprusside (SNP) for 16 hours prior to trans well migration assay. (n = 4), Donors: 2. **(D)** Percentage of CCR5 edited HSCs as measured by ICE analysis of sanger sequencing reads. (n = 3), Donors: 2. Experimental outline for (**E–I**) The FACS purified CD34^+^CD90^+^ HSCs were cultured with vehicle or RUS for 48 hours and 50000 cells were transplanted into 7-8 weeks old female NBSGW mice. 16 weeks post transplantation, different tissues of mice were collected and analysed for the engraftment. Each dot indicates a NBSGW mice. Error bar denotes mean ± SEM. ns; non-significant. *p ≤ 0.05 (Unpaired t test, Two tailed). **(E)** Percentage of human cell engraftment in bone marrow. **(F)** Percentage of human cell engraftment in spleen. **(G)** Percentage of human cell engraftment in peripheral blood. **(H)** Representative FACS plot showing the percentage of mice and human cells in NBSGW mice bone marrow. The percentage in the inset refers to engraftment percentage of human cells calculated as described in *Methods*. **(I)** Multilineage reconstitution [HSPCs (CD34) T cells (CD3), myeloid cells (CD13), B cells (CD19) and monocytes (CD14)] by control and CCR5 edited cells in bone marrow.

To test whether RUS treatment could retain the stemness of sort-enriched CD34^+^CD90^+^ HSCs for CCR5 gene editing, the purified HSPCs were sorted for CD34^+^CD90^+^ HSCs. The CD34^+^CD90^+^ HSCs sorting procedure enriched the most primitive HSCs, marked as CD34^+^CD133^+^CD90^+^CD45RA^-^CD38^-^CD49f^+^ cells (22), by >80% (**Supplementary Figure 2A**). The sorted cells were pre-stimulated with cytokines for 48 hours with or without RUS before gene editing with Cas9 RNP targeting *CCR5*. The CCR5 gene edited cells cultured with RUS displayed increased retention of CD34^+^CD90^+^ cells (**Supplementary Figure 2B** and **Figure 3A**) and high frequency of CD34^+^CD133^+^CD90^+^CD45RA^-^CD38^-^CD49f^+^ HSCs (**Figure 3B**). Consistent with surface expression analysis, the colony formation assay (CFU) showed high frequency of GEMM colonies (**Supplementary Figure 2C**). The RUS treated CD34^+^CD90^+^ cells showed 2-fold increase in CXCR4 expression, a factor crucial for stem cell homing in the bone marrow (**Supplementary Figure 2D**) and thereby resulted in 2-fold greater response towards SDF-1α cytokine ligand (**Figure 3C**). Additionally, sodium nitroprusside (SNP) treatment mediated activation of nitric oxide signaling is shown to increase the CXCR4 expression, transwell migration and homing of HSPCs isolated from umbilical cord blood (36). Remarkably, RUS treatment suffices to improve the SDF1-α mediated transwell migration of the CD34^+^CD90^+^ HSCs at comparable levels as that of SNP treated CD34^+^CD90^+^ HSCs (**Figure 3C**). RUS treatment also showed modest increase in the editing frequency (79% *vs* 60% in vehicle) (**Figure 3D**).

To confirm that RUS treated CD34^+^CD90^+^ HSCs have superior engraftment potential *in vivo*, we transplanted the CD34^+^CD90^+^ HSCs that were cultured for 48hrs with cytokines and with or without RUS into NBSGW mice. The RUS treated cells displayed a higher human cell chimerism in bone marrow (**Figure 3E**), spleen (**Figure 3F**) and peripheral blood (**Figure 3G**) than the vehicle treatment. Strikingly, infusion of 50,000 RUS treated CD34^+^CD90^+^ cells was sufficient to repopulate approximately 56% of mouse bone marrow (**Figure 3H**). The RUS treatment did not alter the multi lineage repopulation capacity of the CD34^+^CD90^+^ cells and we detected lineages such as HSPCs (CD34), T cells (CD3), myeloid cells (CD13), B cells (CD19) and monocytes (CD14) (**Figure 3I**). These results demonstrate that RUS supplementation during culture of HSCs preserves stemness and provides an improvised culture condition for CCR5 gene editing.

### Efficient CCR5 Gene Editing in CD34^+^CD90^+^ HSCs Provides HIV Resistance

**Figure 4:**
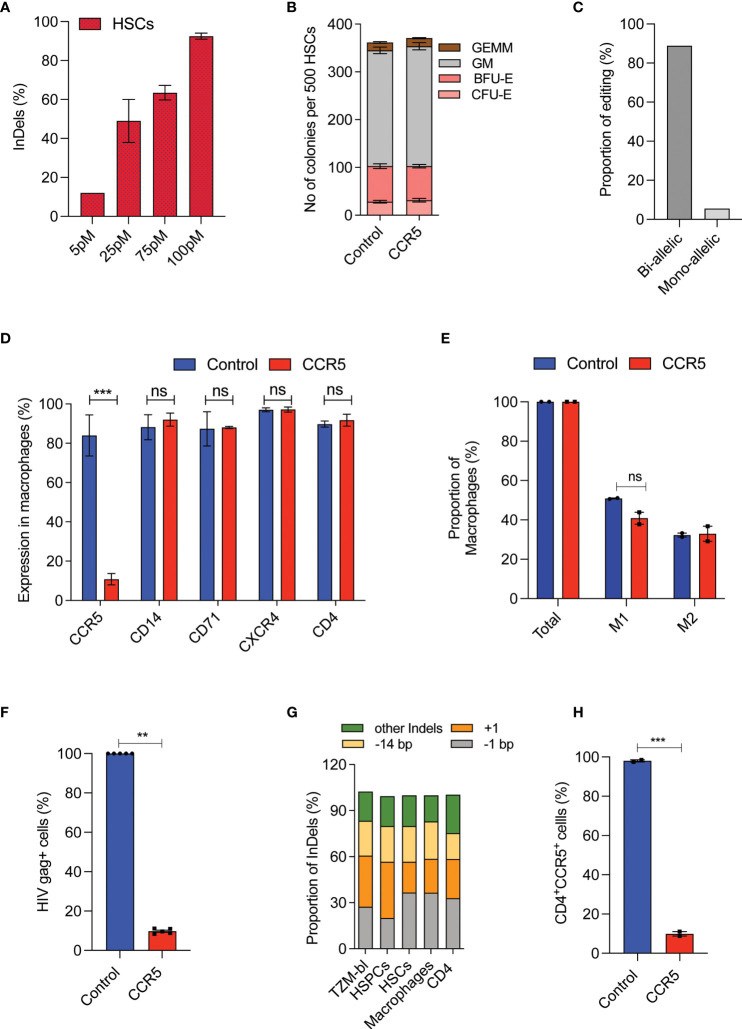
High-frequency *CCR5* editing in CD34^+^CD90^+^ HSCs provide HIV resistance.**(A)** Percentage of *CCR5* editing in HSCs with different doses of Cas9-RNP. (n = 4), Donors: 3. **(B)** Colony forming analysis of control and *CCR5* edited HSCs. n = 6, Donors: 3. **(C)** Percentage of mono and biallelic editing in methocult colonies. CCR5 edited HSCs are single cell sorted in the macrophage differentiation medium and the colonies generated were genotyped. No of colonies analysed: 20. **(D)** Expression pattern of macrophage receptors. The control and *CCR5* edited HSCs were *in vitro* differentiated into macrophages and analysed by flow cytometry for the expression of different markers. (n = 5), Donors: 2. **(E)** Percentage of M1 and M2 macrophage subsets. The control and CCR5 edited HSCs were *in vitro* differentiated into macrophages and analysed by flow cytometry for the expression of total (CD14), M1 (CD14^+^CD80^+^) and M2 (CD14^+^CD206^+^CD163^+^). (n = 2), Donor: 1. **(F)** Percentage of HIV-1 infection in macrophages. The control and *CCR5* edited HSC derived macrophages were infected with HIV-1. HIV Gag^+^ cells were analyzed 6 days post infection using flowcytometry (n = 4), Donors: 2. HIV infection (Gag+ Cells) in control macrophages was normalized to 100. **(G)** Pattern and proportion of Cas9-RNP induced InDels at *CCR5* in TZM-bl cells, HSPCs, HSCs, macrophages and CD4 T cells. (n = 3), Donors: 2. **(H)** CD4 T cells expressing CD4 and CCR5 receptors post gene editing with control or CCR5. (n = 2), Donor: 1. Error bar denotes mean ± SEM. ns, non-significant. **p ≤ 0.01, ***p ≤ 0.001(Unpaired t test, Two tailed).

The CD4 T-cells and CD34^+^HSPCs are the currently explored grafts for CCR5 gene editing. To utilize the CD34^+^CD90^+^ HSCs for CCR5 gene editing, The RUS treated CD34^+^CD90^+^ HSCs were edited for *CCR5* with different concentrations of Cas9-RNP complex and >90% InDels were observed with 100 pmol of RNP (**Figure 4A** and **Supplementary Figure 3B**). The *CCR5* edited cells produced similar number and pattern of multilineage colonies as the control cells in the CFU assay (**Figure 4B**). The edited CD34^+^CD90^+^ HSCs were single cell sorted in methocult medium and the analysis of clonal colonies showed that >85% cells were bi allelic edited cells (**Figure 4C**).

*In-vitro* macrophage differentiation of CCR5 edited CD34^+^CD90^+^ HSCs generated comparable macrophage cell yield (**Supplementary Figure 3C**), expression of respective lineage markers CD14, CD71, CXCR4, and CD4 (**Figure 4D**) and subsets of macrophages (M1 and M2) to that of control (**Figure 4E**). Importantly, 90% of the macrophages lacked the CCR5 expression (**Figure 4D**). The lack of CCR5 expression has not affected the phagocytosis, an important function of macrophages (**Supplementary Figures 3D, E**).

Next, we infected the macrophages with the R5-tropic virus. We observed an active R5-tropic HIV infection in control macrophages whereas >80% of CCR5 modified cells were resistant to infection as determined by HIV gag p24 staining (**Figure 4F** and **Supplementary Figure 3F**). Of note, InDel patterns generated during CCR5 editing of CD34^+^CD90^+^ HSCs were conserved in the TZM-bl cells, CD4 T-cells, HSPCs, and the macrophages (**Figure 4G**). Such InDel pattern in CD4 T cells resulted in CCR5 null CD4 cells suggesting the uniform functional outcome (**Figure 4H**). All these suggest that CCR5 editing in CD34^+^CD90^+^ HSCs generates CCR5 null lineage cells which are HIV resistant and functionally intact.

### A Low Dose of CCR5 Edited CD34^+^CD90^+^ HSCs Is Sufficient to Produce HIV Resistant Immune System

As RUS treatment preserves the engraftment potential of CD34^+^CD90^+^ HSCs, we hypothesized that a low dose of RUS treated CCR5 edited CD34^+^CD90^+^ HSCs could repopulate the mouse bone marrow. We tested this by sorting the CD34^+^CD90^+^ HSCs, cultured it for 48hours with RUS cocktail and gene edited 1x10^5^ CD34^+^CD90^+^ HSCs with Cas9-RNP targeting CCR5 loci or Cas9- tracrRNA as a control. The crRNA less Cas9- tracrRNA control will not induce DNA double strand breaks and thus helps to better understand any gene-editing associated engraftment defect.

**Figure 5:**
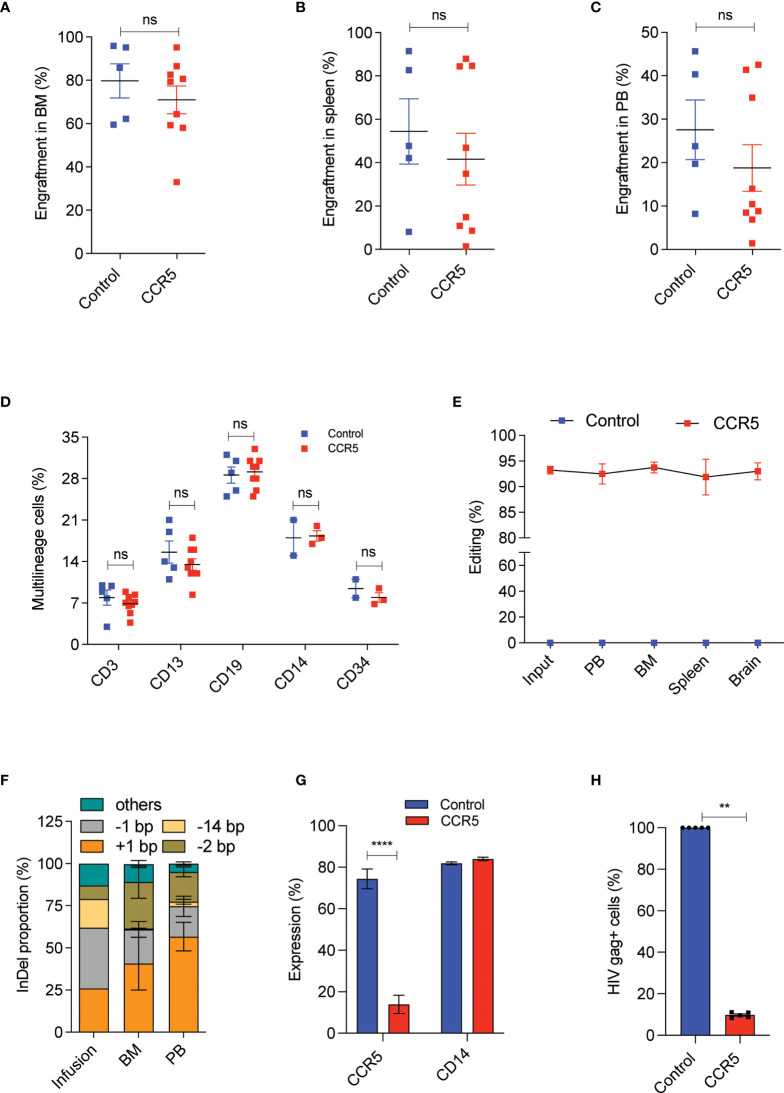
A low dose of CCR5 edited CD34^+^CD90^+^ HSCs generates HIV resistant immune system. Experimental Outline: The FACS purified CD34^+^CD90^+^ HSCs were cultured with vehicle or RUS for 48 hours, electroporated with tracrRNA and Cas9 (control) or Cas9 RNP targeting *CCR5* and approximately 90,000 cells were transplanted into 7-8 weeks old female NBSGW mice. 16 weeks post transplantation, different tissues of mice were collected and analysed for the engraftment. Each dot indicates a mouse. Error bar denotes mean ± SEM. ns; non-significant. **p ≤ 0.01, ****p ≤ 0.0001(unpaired t test, Two-tailed). **(A)** Percentage of human cell engraftment in bone marrow. **(B)** Percentage of human cell engraftment in spleen. **(C)** Percentage of human cell engraftment in peripheral blood (PB). **(D)** Multilineage (T cells (CD3), Myeloid cells (CD13), B cells (CD19), monocytes (CD14) and HSPCs (CD34)) engraftment in bone marrow. **(E)** Persistence of CCR5 edited cells in different tissues (PB = peripheral blood, BM = Bone marrow). (Genomic DNA were isolated from all mentioned tissues and InDels in CCR5 gene is quantified using human CCR5 specific (E2C5-F, E2C5-R) primers. **(F)** Type and proportion of InDels in the infused product and in engrafted cells (BM = bone marrow, PB = peripheral blood). Others refers to InDels with poor read quality. **(G)** Percentage of macrophages with CCR5 and CD14 expression. The engrafted bone marrow cells were differentiated to macrophages *in vitro* and analysed for the expression of CCR5 and CD14 receptors. **(H)** Percentage of macrophages with HIV infection (Gag^+^ cells). The engrafted bone marrow cells were differentiated to macrophages *in vitro* and challenged with HIV-1 virus. 6 days post infection, The HIV infection was measured using flowcytometric staining of HIV-gag protein. HIV infection (Gag+ Cells) in control macrophages was normalized to 100.

At 16^th^ week post transplantation, into NBSGW mice, we observed that the CCR5 edited cells engrafted as efficiently as the control cells with the mean human cell chimerism in bone marrow (**Supplementary Figure 4A** and **Figure 5A**), spleen (**Figure 5B**) and peripheral blood (**Figure 5C**) of about > 70%, 60% and 20% respectively. This analysis indicates that CCR5 gene editing does not affect the engraftment of CD34^+^CD90^+^ HSCs and a dose of 1x10^5^ CD34^+^CD90^+^ HSCs is sufficient to repopulate 70% of the bone marrow. Multilineage analysis in bone marrow showed the formation of myeloid and lymphoid lineages with no lineage bias in the CCR5 edited group (**Figure 5D**).

Tissue trafficking of CCR5^-^ cells is crucial to eradicate the established HIV-1 reservoirs. To test whether CCR5 edited cells can lodge in to different mouse tissues, we analysed the CCR5 editing frequency in the cells recovered from different tissues. InDel analysis showed reconstitution of CCR5 edited cells in bone marrow, spleen, peripheral blood and brain. Importantly, the frequency of gene-editing was maintained from that of the infused product (**Figure 5E**). InDel pattern analysis confirmed that the HSPCs with the prominent +1 and -1 InDels were retained on long-term repopulation, while the cells with 14bp deletion diminished, suggesting that HSPCs with 14bp deletion is not competent for long-term repopulation (**Figure 5F**). Similar loss of 13bp deletion has been reported with gene editing of **γ**-globin promoter (35).

To test the functional proficiency, macrophages were generated from the engrafted human cells and that showed > 90% of cells lacking CCR5 expression in the CCR5 edited group with no defects in macrophage generation or maturation when compared with the control (**Figure 5G** and **Supplementary Figure 4B**). When these macrophages were challenged with infectious R5-tropic HIV, > 80% of cells showed resistance in the CCR5 edited group, consistent with the genotype and CCR5 expression (**Figure 5H**).

## Discussion

In this study, we show that single sgRNA mediated CCR5 gene editing in RUS treated CD34^+^CD90^+^ HSCs is an ideal approach to generate HIV resistant immune system based on the following important observations:

InDels induced by single sgRNA gives HIV resistance as similar as dual sgRNAs approach

Reduced receptors for HIV infection and increased expression of antiviral restriction factors in the CD34^+^CD90^+^ HSCs;

A high frequency of bi-allelic editing in CD34^+^CD90^+^ HSCs;

Infusion of a low dose of RUS treated CD34^+^CD90^+^ HSCs is sufficient to repopulate the bone marrow.

While CCR5 Δ32 allogenic stem cell transplantation has demonstrated the ART independence and undetectable level of viral genome per cell (8), such an effect has not been achieved yet with gene edited autologous HSPCs. The hematopoietic progenitor cells of the HIV infected patients are reported to have various defects including decreased numbers, altered functional characteristics, and defects in the lymphoid lineage (28, 30, 37–39). The defects are probably a result of HIV-1 infection in the HPCs. Studies have shown that HPCs can be infected with HIV-1 and they harbor HIV genome at a frequency similar to CD4^+^ T cells (18, 30). *In vitro* experiments demonstrated that HIV-1 infection in the HPCs triggers apoptosis (28). This provides a possible reason for the peripheral blood cytopenia in the AIDS patients (40). In addition, if HSPCs are used for gene manipulation, reservoirs in the HPCs may reseed the HIV in bone marrow after transplantation. This is counterproductive to the HIV reduction achieved by the conditioning regimens. Also, the activation of latent virus during *ex vivo* culture of progenitor cells poises an additional risk (19, 41). All these findings suggest that HPC fraction in the autologous HSPC graft is not suitable for gene editing of HIV-1 gene therapy.

The recent high throughput and non-human primate transplantation studies have demonstrated that long term HSCs are immunophenotypically marked by CD34^+^CD90^+^ and they could be the ideal target cells for gene manipulation (20, 35). Here, we show that CD34^+^CD90^+^ HSCs are limited with HIV receptor/co-receptor, express increased amount of antiviral restriction factors and exhibit resistance to R5-tropic infection, making the CD34^+^CD90^+^ HSCs ideal target cells for CCR5 manipulation. Our HIV-1 infection studies in the HSPC pool, purified progenitors and HSCs, clearly support the predominant infection of R5 tropic virus in the progenitors but not in HSCs. While all our observations are from mobilized healthy donor HSPCs, infected *ex vivo*, reports from the HIV infected patients showed that the G_0_ fraction of HSPCs lacked any R5 pro-viral DNA, strengthening the use of our approach (27, 38, 39).

The absence of CCR5 receptor and the increased expression of antiviral restriction factors may play a key role in protecting the CD34^+^CD90^+^ HSCs population from HIV-1 infection during pre and post manipulation. SAMHD1 dependent phenomenon demonstrated to restrict HIV infection in macrophages, resting T cells and dendric cells (43). SAMHD1 over expression in the HSCs points that the HSCs protect themselves by a similar mechanism. The antiviral restriction factors in the CD34^+^CD90^+^ HSCs may also limit the HIV infection post transplantation when it encounters the infected stromal cells (44).

Pre-clinical lentiviral gene therapy studies have reported a low lentiviral transduction in the CD34^+^CD90^+^ HSCs when compared with HPC fraction (45, 46). Our observation of higher expression of antiviral factors in the CD34^+^CD90^+^ HSCs explains the reason behind such a low lentiviral transduction.

The complete elimination of CCR5 expression in the lineages by gene editing the CD34^+^CD90^+^ HSCs will be a safer and long-lasting approach as this provides no choice for gp-120 HIV variants to infect hematopoietic cells. CCR5 Δ32 heterozygous genotype showed delayed but not completely prevented HIV-1 infection underlines the need for biallelic CCR5 editing (47).The mathematical modeling predicted that the autologous HSPC graft with an editing efficiency of 76% or greater is required to control the viral rebound (48). Thus, high frequency bi-allelic CCR5 knockout in HSCs is crucial for clinical success. The high frequency of bi-allelic editing (>85%), all InDels being functional in disrupting CCR5 expression and the maintenance of indels post transplantation indicate the clinical potential of our approach. Previous works from pre-clinical studies and clinical trial pointed out a reduction in the CCR5 gene edited cell frequency post transplantation (13, 49–51). However, our CCR5-gene edited cell frequency in the infused product and the engrafted cells are comparable. The elimination of HPC fraction during gene editing has likely contributed to the steady level of gene edited cells post transplantation. Notably, The RUS treatment of HSCs facilitated robust and persisted engraftment *in vivo*.

Manipulating large doses of HSPCs for transplantation is challenging and if the HSPCs carry an infectious virus such as HIV it becomes a daunting task. About 2 million HSPCs are being transplanted in the NBSGW mice to achieve >70% human cell chimerism (52, 53). Our observations are in line with the NHP studies that achieved a high level of chimerism with a 10-fold lower infusion product containing only HSCs (35).

The exclusive usage of HSCs for transplantation is associated with its own limitations (54). The dynamics of gene modified stem cell repopulation suggests that, the steady state hematopoiesis is mediated by the HSCs and are stabilized in about 6-12 months post transplantation (55, 56). Thus, it may take up to a period of 1 year for the complete reconstitution of CCR5^-^ cells in tissues using our approach. Hence, administration of ART for the first year of transplantation is desirable to achieve the full benefit. Studies have also reported a delayed neutrophil reconstitution after transplantation of graft solely containing only the HSCs (35). It will be interesting to see whether the small fraction of HPCs in our graft could potentially provide sufficient numbers of early phase neutrophils. On the other hand, a latest report challenges the bi-phasic hematopoietic reconstitution model and provides evidence that HSCs can also contribute to the early neutrophil recovery (57). Based on this model, CCR5 gene edited CD34^+^CD90^+^HSCs should result in early reconstitution of hematopoiesis with CCR5^-^ cells.

While, the approach can be used both with allogenic and autologous stem cell transplantation, the factors like graft-versus-HIV reservoir and the extensive conditioning used in allogenic stem cell transplantation may help reduce the amount of viral reservoir, independent of gene editing effect (58). However, GvHD could be a big hindrance in using allogenic graft (4). Our strategy will be limited to R5-tropic but not X4 tropic virus as we see a high expression of CXCR4 receptor in CD34^+^CD90^+^ HSCs and this explain the reason for X4 tropic infection in the HSCs (29). While gene editing of HSPCs is observed to be safe in the ongoing clinical trials, the recent pre-clinical observation of chromothripsis in BCL11A enhancer gene edited HSPCs is a matter of concern (59). Therefore, our future studies will be in the direction of characterization of safety profile of CCR5 edited CD34^+^CD90^+^ HSCs.

In summary, we show that CCR5 gene editing in CD34^+^CD90^+^ HSCs provides uninfected and highly engraftable graft for autologous transplantation and presents a safe and highly efficient gene editing approach for HIV gene therapy. Additionally, culture and gene editing of a low dose of cells would facilitate CCR5 gene editing a simplified and cost- effective gene therapy approach.

## Data Availability Statement

The raw data supporting the conclusions of this article will be made available by the authors, without undue reservation.

## Ethics Statement

The studies involving human participants were reviewed and approved by Institute Review Board (IRB), Christian Medical College, Vellore, Tamil Nadu, India. The patients/participants provided their written informed consent to participate in this study. The animal study was reviewed and approved by Institute animal ethical committee, Christian Medical College, Vellore, Tamil Nadu, India.

## Author Contributions

ST designed the study. KVK and ST designed the experiments, analyzed the data, and wrote the manuscript. KVK, JD, VV, AC, PB, MA, AJ, VR, and SR performed the experiments. RK supervised viral studies. AS and GV provided study material and critical inputs. SM and MK provided critical revision of the manuscript. ST acquired the fund. All authors contributed to the article and approved the submitted version.

## Funding

This work was funded by the Department of Biotechnology, Government of India (grant no.BT/PR26901/MED/31/377/2017 and BT/PR38267/GET/119/348/2020). KVK received fund from Department of science and technology, Innovation in Science Pursuit for Inspired Research (DST-INSPIRE-180918). AC is funded by an ICMR-SRF fellowship and PB by CSIR-JRF fellowship.

## Conflict of Interest

The authors declare that the research was conducted in the absence of any commercial or financial relationships that could be construed as a potential conflict of interest.

## Publisher’s Note

All claims expressed in this article are solely those of the authors and do not necessarily represent those of their affiliated organizations, or those of the publisher, the editors and the reviewers. Any product that may be evaluated in this article, or claim that may be made by its manufacturer, is not guaranteed or endorsed by the publisher.

## Supplementary Material

Supplementary Figure 1 HIV receptors in HSPCs, HPCs and HSCs. **(A)** Graphical representation showing sgRNA binding site in CCR5 gene (Nucleotides marked in red showing gRNA targeting region, blue showing Protospacer adjacent motif (PAM) site, shades of blue arrow indicate 246bp deletion when using dual sgRNAs (E2E3) system). **(B)** Representative FACS plot showing the percentage of CD34^+^CD90^+^ cells in unsorted CD34^+^HSPCs and purity of sorted HPCs (CD34^+^CD90^-^cells) and HSCs (CD34^+^CD90^+^ cells). **(C)** Representative FACS plot showing the percentage of HIV receptors (CD4/CCR5) in CD34^+^HSPCs, CD34^+^CD90^+^HSCs and CD34^+^CD90^-^ HPCs. The inset in the bottom plot denotes percentage of cells positive for both CD4 and CCR5.

Supplementary Figure 2 RUS treatment improves the stem cell potential of CD34^+^CD90^+^ HSCs. **(A)** Percentage of highly primitive HSCs (CD34^+^CD133^+^CD90^+^CD45RA^-^CD38^-^CD49f^+^) in HSPCs (unsorted), CD34^+^CD90^+^ HSCs and CD34^+^CD90^-^ HPCs. The cells were analysed immediately after purification or sorting. n = 3, Donor:2. **(B)** Representative FACS plot showing percentage cells expressing CD34^+^CD90^+^ in vehicle and RUS treatment. The sorted CD34^+^CD90^+^ HSCs were cultured with vehicle or RUS for 72 hours before FACS analysis. **(C)** Number of GEMM colonies from CD34^+^CD90^+^ HSCs that are cultured with vehicle or RUS for 3 days. **(D)** Percentage of CD34^+^CD90^+^CXCR4^+^ cells. The CD34^+^CD90^+^ HSCs were sorted and cultured with vehicle or RUS for 3 days. n = 3, Donors: 2. Error bars denotes mean ± SEM, ns, non-significant. *p ≤ 0.05, (Unpaired t test, Two tailed).

Supplementary Figure 3 CCR5 editing in CD34^+^CD90^+^ HSCs generates macrophages with HIV-1 resistance. **(A)** Representative sanger reads from the HSPCs showing intact 32bp sequence which are absent in individuals with delta32 genotype. **(B)** Representative sanger reads from the HSPCs showing intact 32bp sequence which are absent in individuals with delta32 genotype. **(C)** Proliferation kinetics of macrophage progenitor cells from control and *CCR5* edited CD34^+^CD90^+^ HSCs during *in vitro* macrophage differentiation. n = 4, Donors: 2. **(D)** Representative fluorescence micrograph at 10x magnification. The *in vitro* differentiated macrophages were incubated for an hour with fluorescently labelled *E. coli* particle (red). The phagocytosed *E. coli* particle is seen as red colour inside macrophages. (Scale bars were indicated at the right corner of the image. **(E)** Percentage of phagocytosis by control and *CCR5* edited macrophages. Macrophages were incubated with pHrodo Red *E. coli* BioParticles for 1 hr and images were taken with fluorescence microscope and the percentage of phagocytosis was calculated as follows: number cells showing pHrodo Red *E. coli/*number of total cells*100. n = 2, Donor: 1. **(F)** Representative FACS plot showing the percentage of HIV-1 Gag^+^ macrophages from control and *CCR5* edited conditions. Error bars denotes mean ± SEM, ns; non-significant. (Unpaired t test, Two tailed).

Supplementary Figure 4 Low dose of *CCR5* edited HSCs provides robust engraftment in NBSGW mice with CCR5 null macrophages. **(A)** Representative FACS plot showing the percentage of Human and mice cells in different tissues of NBSGW mice after 16 weeks of infusion. (BM - Bone Marrow, PB - Peripheral Blood and Spleen. Inset values denotes percentage of human cell engraftment calculated using formula (% hCD45^+^)/(% hCD45 ^+^ +% mCD45.1^+^) x 100. **(B)** Representative FACS plot showing the percentage of macrophages with CD14 and CCR5. The bone marrow engrafted control and *CCR5* edited cells were *in vitro* differentiated into macrophages and challenged with R5-tropic HIV and analyzed for HIV Gag+ cells as described in *Methods*.
