# Supplementary figures and images for: The CCR5 Gene Edited CD34+CD90+ Hematopoietic Stem Cell Population Serves as an Optimal Graft Source for HIV Gene Therapy
Source: Front Immunol. 2022 Mar 14;13:792684. doi: 10.3389/fimmu.2022.792684 (PMC8963924; doi:10.3389/fimmu.2022.792684)

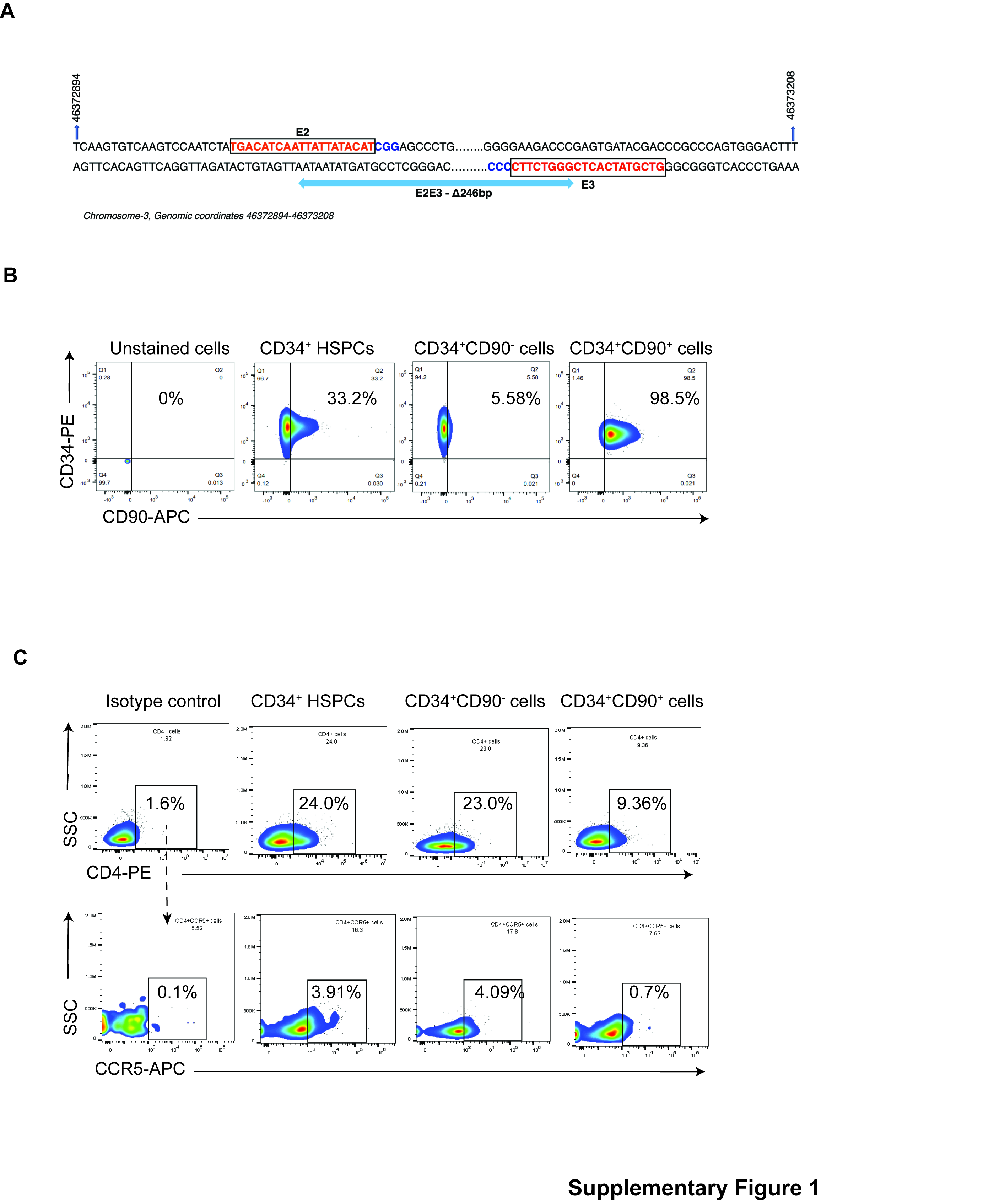

Supplement: Supplementary Figure 1 — HIV receptors in HSPCs, HPCs and HSCs. (A) Graphical representation showing sgRNA binding site in CCR5 gene (Nucleotides marked in red showing gRNA targeting region, blue showing Protospacer adjacent motif (PAM) site, shades of blue arrow indicate 246bp deletion when using dual sgRNAs (E2E3) system). (B) Representative FACS plot showing the percentage of CD34+CD90+ cells in unsorted CD34+HSPCs and purity of sorted HPCs (CD34+CD90-cells) and HSCs (CD34+CD90+ cells). (C) Representative FACS plot showing the percentage of HIV receptors (CD4/CCR5) in CD34+HSPCs, CD34+CD90+HSCs and CD34+CD90- HPCs. The inset in the bottom plot denotes percentage of cells positive for both CD4 and CCR5. [file Image_1.tif]

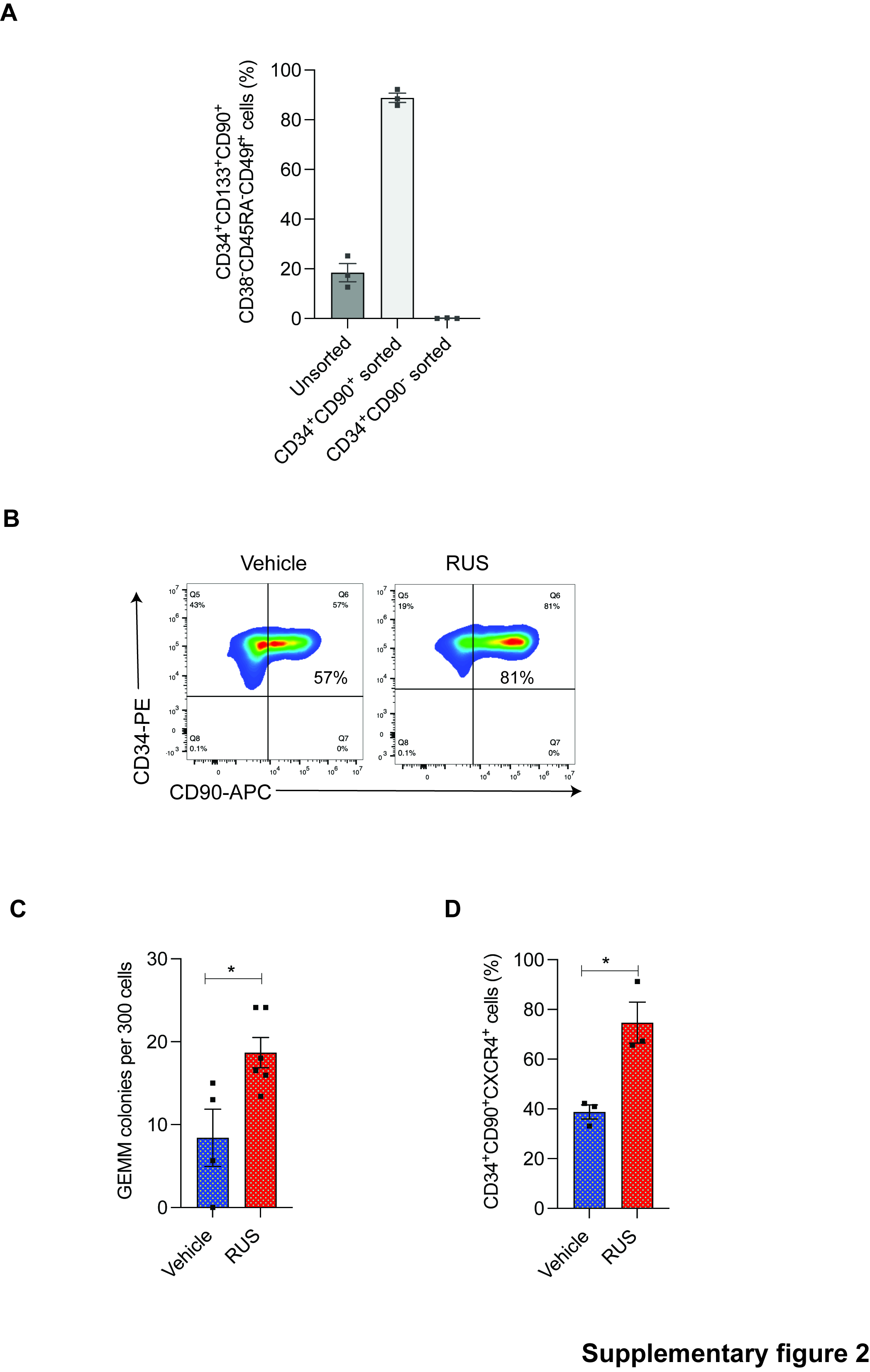

Supplement: Supplementary Figure 2 — RUS treatment improves the stem cell potential of CD34+CD90+ HSCs. (A) Percentage of highly primitive HSCs (CD34+CD133+CD90+CD45RA-CD38-CD49f+) in HSPCs (unsorted), CD34+CD90+ HSCs and CD34+CD90- HPCs. The cells were analysed immediately after purification or sorting. n = 3, Donor:2. (B) Representative FACS plot showing percentage cells expressing CD34+CD90+ in vehicle and RUS treatment. The sorted CD34+CD90+ HSCs were cultured with vehicle or RUS for 72 hours before FACS analysis. (C) Number of GEMM colonies from CD34+CD90+ HSCs that are cultured with vehicle or RUS for 3 days. (D) Percentage of CD34+CD90+CXCR4+ cells. The CD34+CD90+ HSCs were sorted and cultured with vehicle or RUS for 3 days. n = 3, Donors: 2. Error bars denotes mean ± SEM, ns, non-significant. *p ≤ 0.05, (Unpaired t test, Two tailed). [file Image_2.tif]

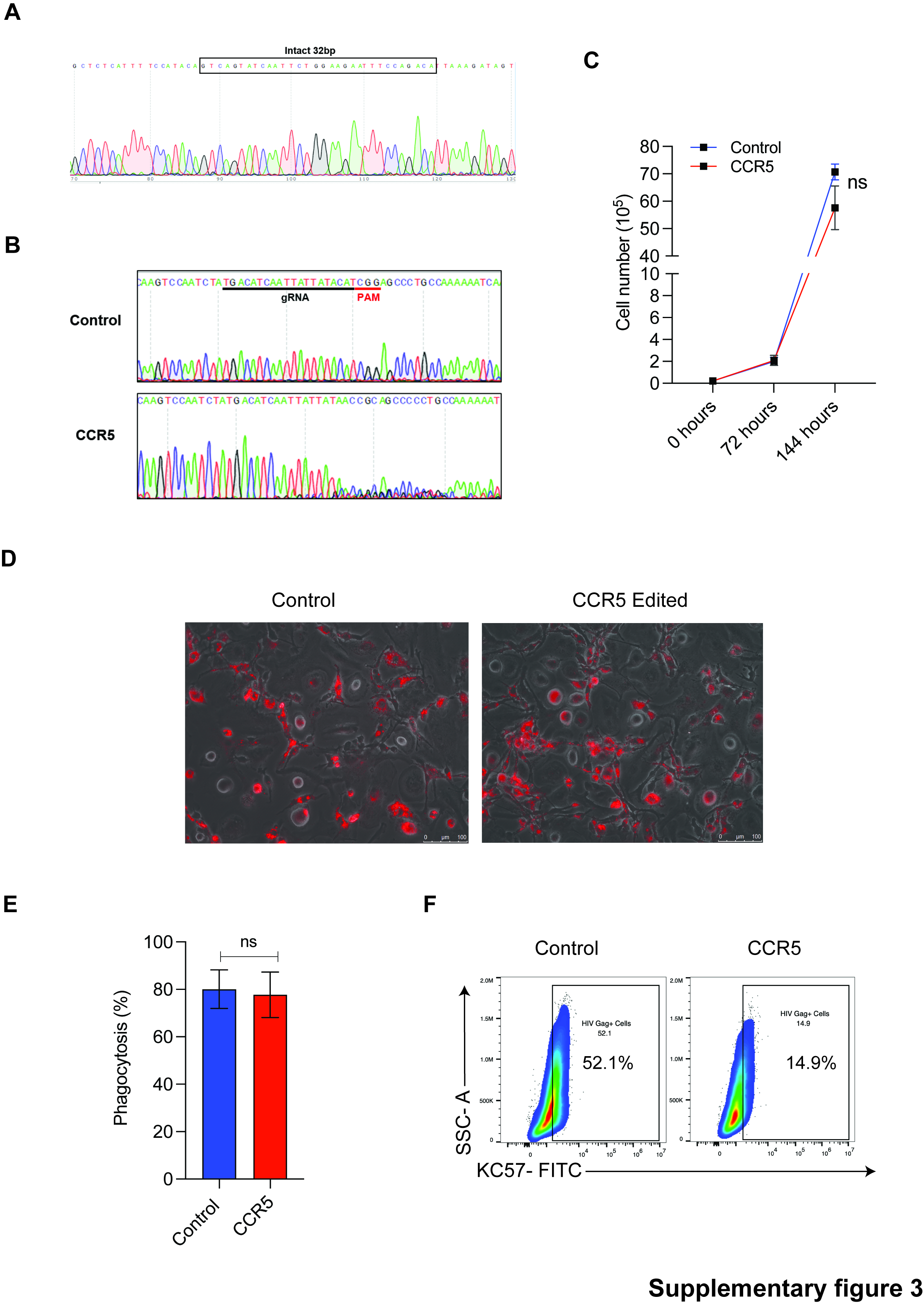

Supplement: Supplementary Figure 3 — CCR5 editing in CD34+CD90+ HSCs generates macrophages with HIV-1 resistance. (A) Representative sanger reads from the HSPCs showing intact 32bp sequence which are absent in individuals with delta32 genotype. (B) Representative sanger reads from the HSPCs showing intact 32bp sequence which are absent in individuals with delta32 genotype. (C) Proliferation kinetics of macrophage progenitor cells from control and CCR5 edited CD34+CD90+ HSCs during in vitro macrophage differentiation. n = 4, Donors: 2. (D) Representative fluorescence micrograph at 10x magnification. The in vitro differentiated macrophages were incubated for an hour with fluorescently labelled E. coli particle (red). The phagocytosed E. coli particle is seen as red colour inside macrophages. (Scale bars were indicated at the right corner of the image. (E) Percentage of phagocytosis by control and CCR5 edited macrophages. Macrophages were incubated with pHrodo Red E. coli BioParticles for 1 hr and images were taken with fluorescence microscope and the percentage of phagocytosis was calculated as follows: number cells showing pHrodo Red E. coli/number of total cells*100. n = 2, Donor: 1. (F) Representative FACS plot showing the percentage of HIV-1 Gag+ macrophages from control and CCR5 edited conditions. Error bars denotes mean ± SEM, ns; non-significant. (Unpaired t test, Two tailed). [file Image_3.tif]

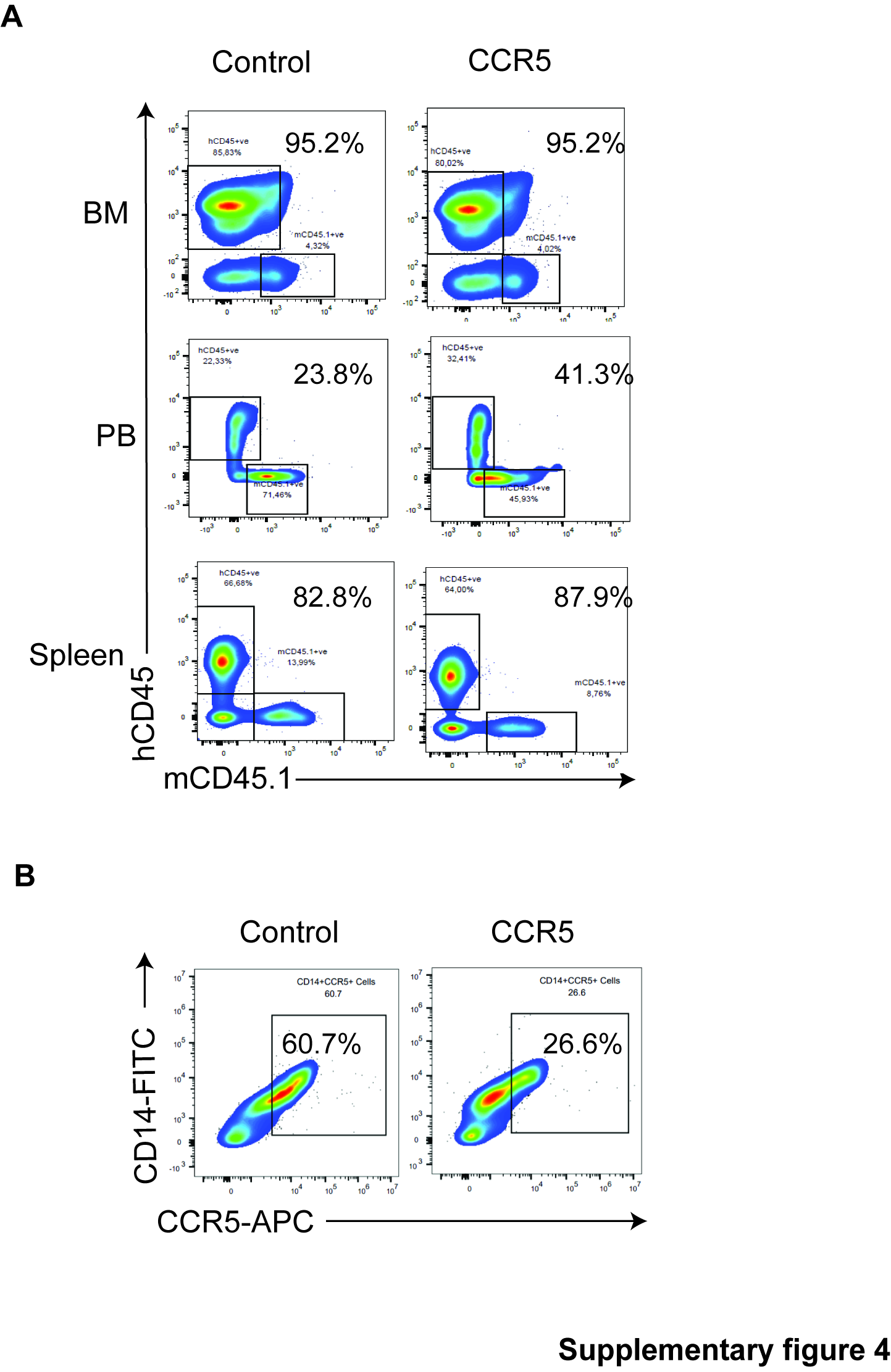

Supplement: Supplementary Figure 4 — Low dose of CCR5 edited HSCs provides robust engraftment in NBSGW mice with CCR5 null macrophages. (A) Representative FACS plot showing the percentage of Human and mice cells in different tissues of NBSGW mice after 16 weeks of infusion. (BM - Bone Marrow, PB - Peripheral Blood and Spleen. Inset values denotes percentage of human cell engraftment calculated using formula (% hCD45+)/(% hCD45 + +% mCD45.1+) x 100. (B) Representative FACS plot showing the percentage of macrophages with CD14 and CCR5. The bone marrow engrafted control and CCR5 edited cells were in vitro differentiated into macrophages and challenged with R5-tropic HIV and analyzed for HIV Gag+ cells as described in Methods. [file Image_4.tif]
