# Supplementary material for: The CCR5 Gene Edited CD34+CD90+ Hematopoietic Stem Cell Population Serves as an Optimal Graft Source for HIV Gene Therapy
Source: Front Immunol. 2022 Mar 14;13:792684. doi: 10.3389/fimmu.2022.792684 (PMC8963924; doi:10.3389/fimmu.2022.792684)
Supplement: Supplementary file 5 [file Table_1.docx]

| **ANTIBODY** | **COMPANY** | **CLONE** | **CAT NO** |
| --- | --- | --- | --- |
| CD45.1 PerCP Cy5.5 | BD | A20 | 560580 |
| CD3 PE Cy 7 | BD | SP34-2 | 557749 |
| CD 90 APC | BD | 5 E10 | 559869 |
| CD13 PE | BD | WM15 | 555394 |
| CD71 FITC | BD | M-A712 | 555536 |
| CD 49f BV421 | BD | GOH3 | 747725 |
| CD 133/1 (AC133) FITC | MACS MILTENYI | AC-133 | 130-113-111 |
| CD16/CD32 (mouse FC block) | BD | 2.4G2 | 553141 |
| CD 33 PE Cy7 | BD | P67.6 | 333946 |
| CD45 APC | BD | HI30 | 555485 |
| CD38 BV786 | BD | HIT2 | 563964 |
| CD34 PE | BD | 8G12 | 348057 |
| CD14 FITC | BD | M5E2 | 555397 |
| CCR5 APC | BD | 2D7 | 561748 |
| CD19 PerCP | BD | SJ25C1 | 340421 |
| CD45 RA PE-CF594 | BD | 5H9 | 565419 |
| CD4 PE | BD | RPA-T4 | 555347 |
| CD4 PerCP | Biolegend | OKT4 | 317431 |
| CD184 PerCP Cy5.5 (CXCR4) | BD | 12G5 | 560670 |
| CD184 APC (CXCR4) | Biolegend | 12G5 | 306509 |
| CD14 BV421 | BD | MɸP-9 | 565283 |
| CD80 FITC | BD | L307.4 | 560926 |
| CD206 APC | BD | 19.2 | 561763 |
| C163 PE CF594 | BD | GHI/61 | 562670 |
| CD64 PE | BD | 10.1 | 561926 |
| HIV core antigen - FITC | Beckman Coulter | KC-57 | 6604665 |
| IgG1(Mouse)-FITC | Beckman Coulter | - | IM0639U |
| PE Mouse IgG1, k isotype control | BD | MOPC-21 | 555749 |
| APC Mouse IgG2a, k isotype control | BD | G155-178 | 555576 |

Table-1: List of antibodies used in this study.
