# Supplementary material for: The CCR5 Gene Edited CD34+CD90+ Hematopoietic Stem Cell Population Serves as an Optimal Graft Source for HIV Gene Therapy
Source: Front Immunol. 2022 Mar 14;13:792684. doi: 10.3389/fimmu.2022.792684 (PMC8963924; doi:10.3389/fimmu.2022.792684)
Supplement: Supplementary file 6 [file Table_2.docx]

| Primer Name | Forward primer | Reverse Primer | Reference |
| --- | --- | --- | --- |
| CCR5 | CAGAGCCAAGCTCTCCATC | AGAGACGCAAACACAGCCA | Current article |
| CCR5 Seq-F | AATGTAGACATCTATGTAGG | - | Current article |
| LEDGF/p75 | TCGACTTCAAAGGATACATGCTG | GAGCTTGTTGCATTGTGACCT | Current article |
| APOBEC3G | TCAGAGGACGGCATGAGACTT | TGGAGCCTGGTTGCATAGAAA | 61 |
| TRIM5alpha | TGCTGGCTTCCAACCTGAT | ACAGAGAGGGGCACAATGAA | 61 |
| Tetherin | GAGTGTCGCAATGTCACCCAT | GGAAGCCATTAGGGCCATCAC | 61 |
| IFITM1 | TGCACAAGGAGGAACATGAG | CTTCCTGTCCCTAGACTTCACG | 62 |
| IFITM2 | GAGCAGGAAGTGGCTATGCT | CTTCCTGTCCCTAGACTTCACG | 62 |
| IFITM3 | CTCAAGGAGGAGCACGAGGT | CTTCCTGTCCCTAGACTTCACG | 62 |
| SAMHD1 | TCACAGGCGCATTACTGCC | GGATTTGAACCAATCGCTGGA | 63 |
| Ubiquitin C | ATTTGGGTCGCAGTTCTTG | TGCCTTGACATTCTCGATGGT | 61 |
| HIV-1 LTR and Gag | CCTCAATAAAGCTTGCCTTGAG | CTTCTATTACTTTTACCCATGC | 64 |
| ERV3 | CATGGGAAGCAAGGGAACTAATG | CCCAGCGAGCAAATACAGAATTT | Current article |
| CCR5 Delta32 seq F | TTGGGGTGGTGACAAGTGTG | - | Current Article |

Table-2: List of Primers used in this study.

**References**

61) Mous K, Jennes W, Camara M, Seydi M, Daneau G, Mboup S,et al.. Expression analysis of LEDGF/p75, APOBEC3G, TRIM5alpha, and tetherin in a senegalese cohort of HIV-1-exposed seronegative individuals. *PLoS One* (2012) **7**: doi:10.1371/journal.pone.0033934

62) Wang B, Lam TH, Soh MK, Ye Z, Chen J, Ren EC. Influenza A virus facilitates its infectivity by activating p53 to inhibit the expression of interferon-induced transmembrane proteins. *Front Immunol* (2018) **9**: doi:10.3389/fimmu.2018.01193

63) Sommer AFR, Rivière L, Qu B, Schott K, Riess M, Ni Y, , et al. Restrictive influence of SAMHD1 on Hepatitis B Virus life cycle OPEN. *Scientific reports* (2016) doi:10.1038/srep26616

64) MacNeil A, Sankalé J-L, Meloni ST, Sarr AD, Mboup S, Kanki P. Genomic Sites of Human Immunodeficiency Virus Type 2 (HIV-2) Integration: Similarities to HIV-1 In Vitro and Possible Differences In Vivo. *J Virol* (2006) **80**:7316. doi:10.1128/JVI.00604-06
